# Scalable, ultra-resistant structural colors based on network metamaterials

**DOI:** 10.1038/lsa.2016.233

**Published:** 2017-05-05

**Authors:** Henning Galinski, Gael Favraud, Hao Dong, Juan S Totero Gongora, Grégory Favaro, Max Döbeli, Ralph Spolenak, Andrea Fratalocchi, Federico Capasso

**Affiliations:** 1 John A. Paulson School of Engineering and Applied Sciences, Harvard University, Cambridge 02138, USA; 2 Laboratory for Nanometallurgy, ETH Zurich, Vladimir-Prelog-Weg 1-5/10, Zurich 8093, Switzerland; 3 PRIMALIGHT, King Abdullah University of Science and Technology (KAUST), Thuwal 23955-6900, Saudi Arabia; 4 Anton Paar TriTec SA, Peseux CH-2034, Switzerland; 5 Ion Beam Physics, ETH Zurich, Otto-Stern-Weg 5, Zurich 8093, Switzerland

**Keywords:** nanophotonics, plasmonics, structural colors

## Abstract

Structural colors have drawn wide attention for their potential as a future printing technology for various applications, ranging from biomimetic tissues to adaptive camouflage materials. However, an efficient approach to realize robust colors with a scalable fabrication technique is still lacking, hampering the realization of practical applications with this platform. Here, we develop a new approach based on large-scale network metamaterials that combine dealloyed subwavelength structures at the nanoscale with lossless, ultra-thin dielectric coatings. By using theory and experiments, we show how subwavelength dielectric coatings control a mechanism of resonant light coupling with epsilon-near-zero regions generated in the metallic network, generating the formation of saturated structural colors that cover a wide portion of the spectrum. Ellipsometry measurements support the efficient observation of these colors, even at angles of 70°. The network-like architecture of these nanomaterials allows for high mechanical resistance, which is quantified in a series of nano-scratch tests. With such remarkable properties, these metastructures represent a robust design technology for real-world, large-scale commercial applications.

## Introduction

Billions of years ago, green algae originated life, changing the face of the earth from gray to green and paving the way for the life forms we see today^1^. Since then, living organisms have extensively used color for a variety of purposes, ranging from communication to self-defense, from reproduction to camouflage^2^. The enormous variety of colors, such as the sapphire blue wings of the Morpho butterfly^3, 4^ and the thermochromic coloration of the chameleon^5^, has stimulated the interest of researchers dating back to seventeenth century, when Hooke theorized about the origin of color in the brilliant feathers of peacocks and ducks^6^. Many of these colors do not originate from pigments or dyes but are ‘structural’, resulting from the interaction of light with self-assembled structures of living organisms^4, 7, 8^.

The engineering of structural colors from artificial photonic structures has attracted conspicuous interest in research due to the many applications that can potentially be opened by this technology^5, 9, 10, 11, 12, 13, 14, 15, 16, 17^. Structural colors based on photonic crystals and metamaterials have been explored, showing very promising results, including the possibility to create colors at the diffraction limit^15^. A major challenge is overcoming the problems of limited scalability and lack of robustness, which affect the real-world applicability of photonic crystals and classical metamaterials. It is therefore highly desirable to investigate new approaches that can transform these initial breakthroughs into real-world applications.

In the following, we describe a new biomimetic material that overcomes the aforementioned challenges, introducing a new type of structural coloration that is highly scalable and extremely robust. This nanomaterial takes inspiration from subwavelength nanoscale networks identified in the feathers of *Cotinga maynana*, a South American bird^18^. The non-iridescent blue color of the feathers is produced by an aperiodic nanoporous keratin network with a typical feature size smaller than 200 nm. This lightweight network has extraordinary optical properties that cannot be explained by classical Rayleigh/Mie scattering and are strongly related to the short-range order of the nano-network of the barbs^19^. The interaction of light waves with complex materials has already been reported to have a series of fascinating dynamics, ranging from energy harvesting to ultra-dark nanomaterials and beyond^7, 11, 20, 21, 22, 23, 24, 25, 26, 27^. Taking inspiration from the *Cotinga maynana* feathers as an example in nature of a network-based optical nanomaterial, we create complex nano-photonic structures that combine a cellular metallic network^28, 29^ with subwavelength coatings made by lossless dielectrics. This material combination provides significant advantages for real-world applications: it is suited for large-scale fabrication and is lightweight and mechanically robust, combining the high-yield strength to low density ratio of a cellular metallic network with the resistance to wear that alumina offers^30^. Optically, the interface of such a metallic nanoscale network and the lossless dielectric can be considered as electromagnetically ‘weakly’ rough and an inhomogeneous mixture of dielectric/metal and dielectric/air regions. In this scenario, the component of the wavevector parallel to the interface is not conserved, resulting in a highly spatially dependent electromagnetic response. Taking advantage of such a complex light–matter interaction, we illustrate here how to create colors with remarkable properties.

## Materials and methods

### Sample preparation and characterization

PtYAl layers of 300-nm thickness were deposited at room temperature by magnetron co-sputtering onto SiN_*x*_/Si substrates that were pre-cleaned using isopropanol and acetone. Subsequently, the films were dealloyed in 4 M NaOH at room temperature for 60 s and then rinsed with deionized water. The morphological analysis of the samples was studied via scanning electron microscopy assisted by focused ion beam etching (FIB). The compositional analysis was performed by Rutherford backscattering spectrometry. Detailed information is given in the Supplementary Information. In this work, the Savannah atomic layer deposition (ALD) from Ultratech/Cambridge NanoTech (Waltham, MA, USA) was used to deposit Al_2_O_3_ coatings on the dealloyed metal nanowire networks. During the ALD deposition of Al_2_O_3_, a pulse time of 0.15 s and a purge time of 30 s for both trimethylaluminium and water were used. The base pressure was 500 mTorr, and the working temperature was 250 °C. The growth rate was ~0.1 nm per cycle. For creating colored graphic arts, the 60-nm-thick Al_2_O_3_ film was deposited via radio frequency (RF) sputtering at room temperature using a sputtering tool (AJA International, Scituate, MA, USA). The electromagnetic reflectance of the coated samples was measured using a variable-angle spectroscopic ellipsometer from J.A. Woollam Co. (Lincoln, NE, USA) and a NanoCalc thin film reflectometry setup (Ocean Optics Inc., Dunedin, FL, USA). The dielectric constant of the Al_2_O_3_ coating deposited by ALD was determined using a Cauchy model by analyzing a 53-nm-thick Al_2_O_3_ coating deposited on a Si wafer. The scratch tests were performed using an Anton Paar TriTec Nano Scratch Tester (Anton Paar TriTec SA, Peseux, Switzerland).

### Finite-difference time-domain (FDTD) simulations

**Figure 1:**
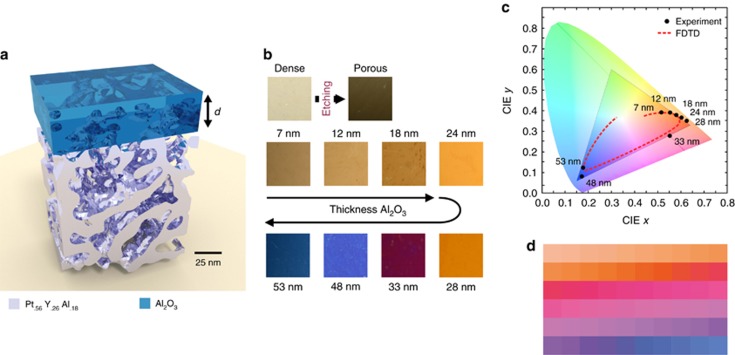
Observation of structural colors in random metallic networks with subwavelength dielectric coatings. (**a**) Schematic illustration of an Al_2_O_3_-coated PtYAl nanomaterial, based on a 3D reconstruction of a completely dealloyed PtYAl thin film obtained via FIB-assisted thin film tomography. (**b**) Photographs of deposited, dealloyed and Al_2_O_3_-coated PtYAl metamaterial networks, illustrating the formation of vibrant colors and the continuous color change with increasing coat thickness. The photographs were taken under illumination from ceiling lights. Each image is 2 × 2 mm^2^. (**c**) Experimental and FDTD simulated structural color reported in a standard CIE 1931 (*x, y*) space, depicting the chromaticity visible to the average person. The RGB color space is marked by the triangle area. The chromaticity is calculated directly from reflectance spectra obtained either experimentally (circles markers) or by FDTD simulations (dashed line). The edges of the tongue-shaped plane correspond to color values of maximal saturation. (**d**) Color palette calculated by FDTD simulations for increasing thickness of Al_2_O_3_. 3D, three-dimensional; RGB, red green blue.

Numerical simulations were carried out using our parallel code NANOCPP, which is a highly scalable (up to hundreds of thousands CPU) Maxwell equation solver, able to include dispersive materials with arbitrary dispersion curves^20^. To build a realistic model for our sample, we considered a metallic structure whose profile was extracted from the morphological analysis of the samples (FIB) shown in Figure 1a. The dispersion parameters of the various materials were taken from direct measurements. Light impinging on the sample was simulated within the transmitted field/scattered field formulation^20^, which allows the detailed modeling of plane wave input excitations on the samples.

## Results and discussion

### Material design and color characterization

We selected dealloying to assemble a nanoscale metallic network with controllable features. This method, first proposed by Raney to synthesize metal catalysts^31^, utilizes the selective dissolution of the less noble constituent of an alloy during wet etching. In our experiments, 300-nm-thick Pt_.14_Y_.06_Al_.80_ thin films were deposited on an amorphous Si_3_N_4_/Si substrate. Although immersing the film in a 4 M aqueous solution of NaOH for 60 s, the less noble Al in the Pt-alloy thin film is subsequently removed, and the remaining metal reorganizes into a network with an open porosity. Characteristic geometrical features of the network can be altered by changing the etching time, the etchant concentration or the initial composition of the thin film^32, 33, 34, 35, 36^.

In a second step, the nanomaterial is coated with an ultra-thin layer of Al_2_O_3_ using ALD. The coating thickness is increased stepwise in a range from 7 to 53 nm. We characterized the growth of the subwavelength Al_2_O_3_ coatings by Rutherford backscattering spectroscopy and FIB-assisted scanning electron microscopy (see Supplementary Information). A three-dimensional image of the Pt_.56_Y_.26_Al_.18_ network, experimentally obtained using FIB thin film tomography, is displayed in Figure 1a.

In a final series of experiments, we characterized the optical response of the network metamaterial for different thicknesses of the dielectric layer Al_2_O_3_. These experiments unveiled a very interesting mechanism of structural coloration from the nanowire network, as shown in Figure 1b. By changing the coating thickness, we observed the formation of a multitude of colors spanning from yellow, orange and red to, finally, blue. The same physical effect with the optical response blue-shifted and smaller color range was observed for a Pt-Al network (see Supplementary Information and Supplementary Fig. S9). Conversely, when the same coatings were deposited on a dense PtYAl metal thin film, no particular color was produced (see Supplementary Information and Supplementary Fig. S5). The colors observed in the metallic network were saturated and go even slightly beyond the red green blue gamut in the CIE chromaticity diagram (Figure 1c).

To illustrate that these colors were consistently observed by varying Al_2_O_3_ layer thickness, we compared experimental results with theoretical predictions based on finite-difference time-domain (FDTD) simulations. For the latter, we used a two-dimensional section of the FIB tomography of the sample illustrated in Figure 1a. Our FDTD simulations, shown in Figure 1c as a dotted line, reproduced the experimental results well, confirming the possibility of achieving such a large variety of colors by tuning the thickness of the Al_2_O_3_ layer. Figure 1b shows experimental images of samples characterized by different thicknesses of Al_2_O_3_. Remarkably, despite the existence of the metallic nanoscale network below the Al_2_O_3_ layer, the samples demonstrated a highly uniform color in all different configurations. A comparison with FDTD calculations is provided in Figure 1d, which illustrates the color palette that can be observed when the thickness of Al_2_O_3_ increases.

**Figure 2:**
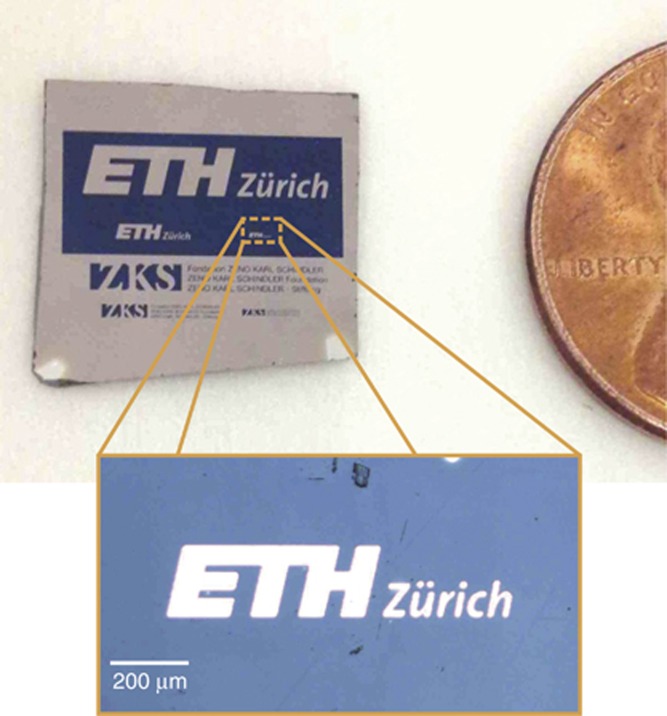
Examples of different graphic arts designs with structural colors from metamaterial networks. Photograph and optical micrograph of a colored graphic art designed by combining a RF-sputtered Al_2_O_3_-coated network metamaterial and photolithography. The inset shows an optical micrograph illustrating a detail of the graphic art and the uniformity of the color.

To emphasize that the structural coloration in these nanoplasmonic structures can be achieved by various deposition techniques, we also fabricated a structural colored graphic arts by using physical vapor deposition. Figure 2 depicts an example created by combining a dealloyed network metamaterial with an RF-sputtered 60-nm-thick Al_2_O_3_ coating and photolithography using a Heidelberg *μ*PG501 optical direct writing system. The bicolored graphic art combines a highly uniform structural color (blue) with a metallic white color (dense film). The material choice for the coating layer is not limited to Al_2_O_3_ a lossless dielectric. Dielectric coatings with and without losses could, in principle, be used to alter the plasmonic response and finally change the structural coloration. Another approach to altering the color impression, especially its saturation, is to change the number of trapping sites within the network metamaterial, for example, by reducing the metamaterial thickness.

### Robustness of structural colors from metamaterial networks

**Figure 3:**
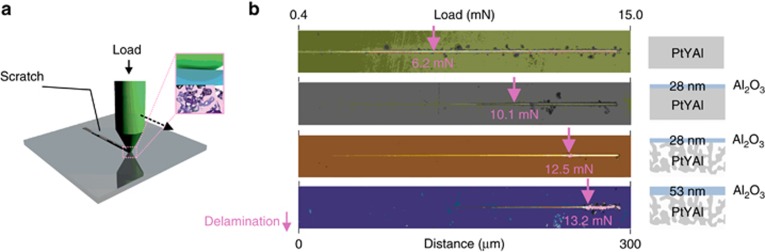
Wear properties of the structural colors. (**a**) Schematic illustration of the basic principle of the scratch testing technique. A diamond stylus is used to scratch the film with progressively increasing load. (**b**) Optical micrographs of progressive load scratches (0.4–15 mN) on a dense PtYAl thin film with and without coating, and PtYAl nanoscale networks coated with 28- and 53-nm-thick Al_2_O_3_, respectively. The critical load characterizing the adhesion failure of the films is indicated by a pink arrow.

**Figure 4:**
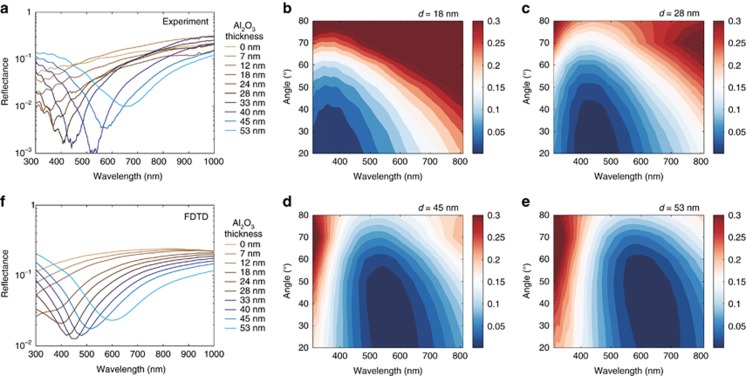
Optical properties of the network metamaterials: reflectivity spectra. (**a**) Experimental normal incidence reflectance spectra as a function of the Al_2_O_3_ coating thickness, *d*. (**b**–**e**) Experimental reflectance spectra of nanoporous PtYAl thin films coated with 18-, 28-, 45- and 53-nm Al_2_O_3_, respectively, as a function of the incidence angle (20°–85°). The value of reflectance is indicated by the color bar. (**f**) FDTD-calculated normal incidence reflectance spectra as function of the Al_2_O_3_ coating thickness, *d.*

To quantify the mechanical robustness of these colors, we resort to nano-scratch resistance testing (Figure 3a), which is an ideal technique to characterize the adhesion failure of coatings. A detailed description of the experimental procedure we used is given in the Supplementary Information. Figure 3b reports optical micrographs of four representative nano-scratch tests. The wear resistance of a dense PtYAl film with and without a 28-nm-thick Al_2_O_3_ coating is compared with a porous nanoscale Pt network coated with 28 and 53 nm of Al_2_O_3_, respectively. The critical load causing delamination of the coated network metamaterial is almost two times higher than the corresponding dense metallic film (Figure 2b) and 20% higher than the dense metallic film coated with 28-nm-thick Al_2_O_3_. Considering the 53% porosity in the nanoscale network, the observed increase in wear resistance is remarkable and indicates an enhanced strength-to-density ratio^37^ corresponding to a significant reduction of overall weight of the coating. Figure 4 illustrates *s*-polarized reflectivity spectra at normal (Figure 4a) and oblique (Figure 4b–4e) incidence for different alumina coating thickness. Figure 4a demonstrates that the formation of colors originates from a large red shift of the reflectivity response of the nanomaterial, observed when the Al_2_O_3_ layer changes thickness. The corresponding FDTD results are reported in Figure 4f. FDTD simulations quantitatively reproduce well the experimental results, confirming the principal role of the Al_2_O_3_ coating layer in red-shifting the spectral response of the material. A small variation of only 30 nm in the Al_2_O_3_ thickness shifts the reflectivity minimum of ~350 nm. Reflectivity spectra of the material are stable and do not show significant variations up to incident angles of 70°, which still provide reflectivity minima as low as <1% (Figure 4b–4e). The mean angular dispersion of the reflectance minimum has been determined from the reflectance spectra obtained by ellipsometry. The mean angular dispersion is independent of the coating thickness and the reflectance minimum blue shifts with −1.0±0.3 nm per degree. These experiments show that the structural colors observed in Figure 1 are non-iridescent, that is, robust against large changes of the incident angle.

### Structural coloration from localized surface states in complex epsilon-near-zero (ENZ) materials

**Figure 5:**
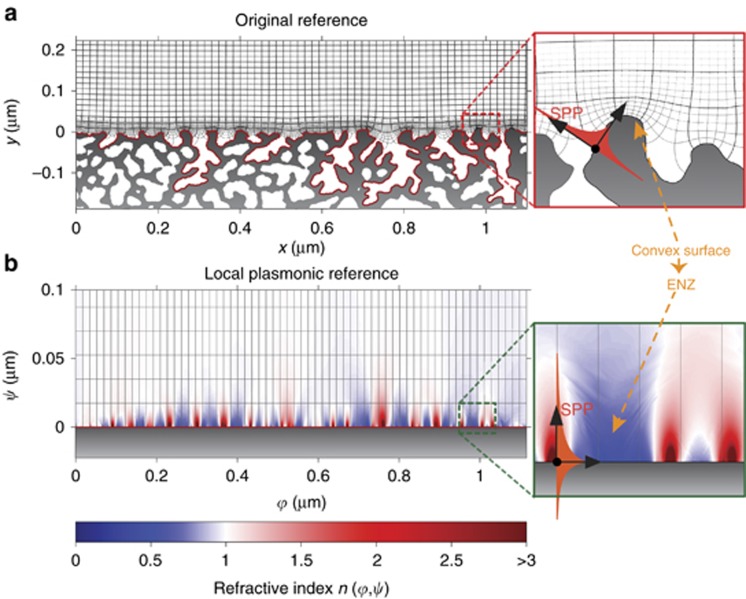
Generation of an equivalent ENZ material in the metallic nanowire network of Figure 1a. (**a**) 2D cross-section profile of the metallic nanowire network, as obtained from experimental FIB images of polished samples (Figure 1a). When light impinges on this structure, it excites the propagation of SPP waves, which move along the complex surface of the metal (**a**, inset). This motion is conveniently described in a curvilinear reference (*φ*, *ψ*), which provides a conformal map of the metallic surface of the sample (solid red line). In the transformed space, (*φ*, *ψ*) (**b**), SPP waves appear to propagate inside an inhomogeneous material with refractive index, *n*(*φ*, *ψ*), on the line at *ψ*=0 (**c**, inset). The material, *n*(*φ*, *ψ*), models the effects of the metallic geometry of **a**, which is flattened out in transformed space, (*φ*, *ψ*). The two systems of **a** and **b** are exactly the same for light propagation. The equivalent structure of **b** demonstrates a complex network of ENZ structures (**b**, dark blue area), which are created by points of convex metallic curvature (right inset). 2D, two-dimensional.

In this section, we analyze in more detail the mechanisms by which structural colors are created and observed in the metallic network of Figure 1. When polychromatic light impinges on the structure of Figure 1a, the interaction between light and matter generates surface plasmon polaritons (SPP)^38^, which are surface waves localized at the metal-dielectric interface of the structure^7^. In our samples (Figure 5a), the motion of SPP develops along complex trajectories in space due to a strongly disordered metallic profile, (Figure 5a, inset). It is convenient to study this motion in a new curvilinear system, whose axes are parallel to the spatial trajectories of SPP. To this extent, we introduce a new set of coordinates, (*ψ*, *φ*), which are conformal to the disordered surface of the metal. Figure 5a shows how these coordinates appear in the original space. *(x,y)*, whereas Figure 5b shows how the original structure appears in the space (*ψ*, *φ*), which we identify as the ‘plasmonic reference’. In the plasmonic reference, the motion of the surface plasmons is extremely simple and composed of straight lines at *ψ*=0 (Figure 5b, inset). When we change spatial coordinates in any electromagnetic system, Maxwell equations remain invariant if we introduce an inhomogeneous refractive index distribution that makes the two reference systems equivalent^39, 40^. The pseudocolor plot in Figure 5b shows the spatial distribution of the inhomogeneous index, *n*(*ψ*, *φ*), computed by using transformation optics (see Supplementary Information). The index, *n*(*ψ*, *φ*), is associated with the coordinate transformation introduced in Figure 5b and acts as a counterpart of the metallic geometry of Figure 5a, which does not exist in Figure 5b, as the metal surface is flattened out. The two structures of Figure 5a and 5b are exactly equivalent: when light propagates in one or another, it follows the same dynamics. This is an exact result of Maxwell equations that contains no approximation. This result also implies that when light impinges on the structure of Figure 5a, it happens to propagate in the medium of Figure 5b. The calculation of a conformal grid for the disordered surface of Figure 5a requires a new formulation of optical conformal mapping, which we recently developed, and allows for the generation of conformal grids for arbitrary structures with arbitrary-large numerical precision. This approach is relatively involved, and it will be discussed in a future work.

**Figure 6:**
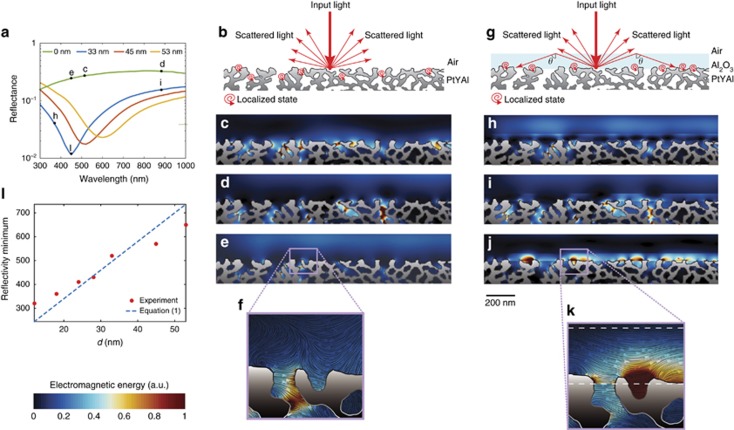
Mechanisms of structural color formation in the PtYAl cellular network. (**a**) Normal incidence reflectance spectra obtained from FDTD simulations of the nanoscale Pt network of Figure 1a with different thickness of Al_2_O_3_. (**b**–**f**) Analysis of the case with no Al_2_O_3_ deposited on top of the metal, while in (**g**–**k**), summary of the results for an Al_2_O_3_ layer of 33 nm. (**b** and **g**) A pictorial illustration of light–matter interactions with the sample, without **b** and with **g** Al_2_O_3_. In the presence of Al_2_O_3_, a portion of scattered waves are reflected back in the Al_2_O_3_ layer, thus creating an energy flow in the coating layer and a resonant coupling with ENZ regions located in the Al_2_O_3_. (**c**–**e** and **h**–**j**) FDTD-calculated spatial energy distributions in the structure by considering an input wavelength indicated by the corresponding letter in **a**. Energy distributions are averaged over one optical cycle at steady state. (**f** and **k**) A zoomed view of the pink area of **e** and **j** and illustrates the electromagnetic energy flow in the structure (arrow colored lines). The flow is superimposed with the corresponding averaged spatial energy distribution. (**l**) Comparison of the reflectivity minimum shift observed in experiments (Figure 4) with theoretical predictions based on the model illustrated in **g**.

The plasmonic reference of Figure 5b illustrates in clear form the effects of disorder, which introduce a strong modulation of the refractive index in the proximity of the metallic surface at *ψ*=0, generating a network of epsilon-near-zero (ENZ) regions, separated by areas of high refractive index (Figure 5b). As observed in the insets of Figure 5a and 5b (dashed lines), ENZ regions are created in the points where the metallic surface is convex, whereas high dielectric permittivities originate in the points where the surface is concave. When waves propagate into an ENZ material, the phase velocity diverges, thus creating standing waves with infinite wavelengths^41, 42, 43^. When SPP waves propagate in the nanowire network of Figure 5a, they ‘see’ the equivalent medium illustrated in Figure 5b and become trapped in the ENZ regions, thereby generating a set of quasi-localized states. We illustrated these dynamics by a series of FDTD simulations. Figure 6a presents a magnified version of Figure 4a, showing FDTD-calculated reflectivity spectra for different thicknesses of the Al_2_O_3_ layer. FDTD results corresponding to different combinations of alumina thicknesses and input wavelengths are summarized in Figure 6b–6j. When light impinges on the disordered metallic structure (Figure 6b), some energy is scattered back, generating components along all directions in space, whereas the remainder is coupled into SPP waves. As illustrated in Figure 6c–6e, which show FDTD-calculated electromagnetic energy density distributions, SPP waves are completely localized in the proximity of different convex points of the surface, exactly where the ENZ regions are formed. FDTD simulations show that different wavelengths are trapped in different ENZ regions of the metal, demonstrating that the ENZ network formed in Figure 5b does not possess a particular length scale and that it traps equivalently all input wavelengths. The absence of a characteristic scale is expected from the strongly disordered surface modulation of the sample, which possesses an abundant variety of different curvatures (Figure 5a) and therefore of ENZ regions with different extensions (Figure 5b). These ENZ regions trap polychromatic light very efficiently, as observed from the flat reflectivity response of Figure 6a (solid green line). To further characterize the energy propagation in the structure, we also plotted the flow of electromagnetic energy in the structure, computed from the Poynting vector of the electromagnetic field (Figure 6f). This is represented with a specific line integral convolution technique, which clearly visualizes the energy flow, characterized by complex patterns with a nontrivial vorticity.

When we deposited a small layer of Al_2_O_3_ on top of the metal, the scattering dynamics changed abruptly (Figure 6g). In this situation, a portion of scattered wavevectors were reflected inside the alumina layer, thus generating a series of additional scattering events in the Al_2_O_3_. Wavevectors propagating at an angle, *θ*, (see Figure 6g) larger than the critical angle, 
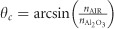
, formed by the interface of air and alumina were totally reflected back and do not radiate energy outside the alumina, surviving the dynamics for many scattering events. These components existed at any given thickness of alumina, as the critical angle depends only on the difference in the refractive index between alumina and air. Reflected wavevectors create a flow of energy in the layer of Al_2_O_3_, inducing a preferential localization of SPP inside ENZ regions that exists within the film of Al_2_O_3_. This process is clearly illustrated in Figure 6h and 6i, which shows the presence of a resonant coupling around the wavelength of 425 nm (Figure 6j). Resonant light localization in ENZ regions within the alumina layer is observed in Figure 6i, which demonstrates light trapping at the wavelength of 425 nm at different points of convex metallic curvature located inside the Al_2_O_3_. Figure 6h and 6i; Supplementary Fig. S7 show electromagnetic energy distributions calculated from the resonance, demonstrating that outside the reflectivity minimum located around the wavelength of 450 nm, no surface localization is formed, and no energy is trapped in the alumina layer. Figure 6k presents line integral convolution images of the Poynting flux clearly showing the flux of energy originated inside the Al_2_O_3_, from the light backscattered from the random metallic surface of the sample. By using arguments from wave theory and scale invariance of Maxwell equations (see Supplementary Information), we obtained a simple relationship for the wavelength shift, Δ*λ*(Δ*d*), as a function of the thickness variation of alumina, Δ*λ*,


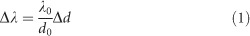


where *λ*_0_ is the wavelength of a reflectivity minimum corresponding to a coating thickness, *d*_0_. Figure 6l compares experimental measures with the results of Equation (1). By applying experimental values for both *λ*_0_ and *d*_0_, we obtained a coefficient *λ*_0_/*d*_0_ ≅ 12, which implies a wavelength shift of 12 nm for every 1 nm increment of coating thickness. The results of Equation (1) show a good agreement with experimental results, predicting the large red shift that is the basis of the structural colors formed in the system.

## Conclusions

We have experimentally demonstrated a new design concept to create robust and saturated structural colors in metasurfaces composed of metallic nanowire networks with ultra-thin, lossless dielectric coatings. Using a combination of analytical and numerical techniques, we illustrated that these colors are the result of the resonant coupling of light with surface plasmons that are localized in equivalent ENZ regions formed in the metallic network. This mechanism is not constrained for large angles as high as 70°, allowing for efficient trapping of light over a broad wavelength range in the visible region. The combination of mechanical robustness and color saturation in an extremely lightweight structure makes these structural colors suitable for real-world industrial applications, such as automotive vehicles or airplanes, for which the weight is directly related to the fuel economy. As discussed in the introduction, achieving a scalable fabrication is a key problem in structural color printing. On the basis of our experiments, it is evident that our metasurfaces have shown a wide color capability without the need for electron beam lithography or other complex fabrication procedures. Our structures, in fact, are based on simple wet-chemistry and coating technologies, which can produce robust colors on large spatial scales. In addition to such fundamental advances, our design concept has the potential to enrich the application of metasurfaces to areas in which large active regions are mandatory, such as efficient light trapping layers in photovoltaic cells. Although a deeper discussion of this topic is beyond the scope of this paper, we can introduce some important points. On the basis of our theory and experiments, we demonstrated that it is possible to control the response of an optical material by ‘engineering’ the connectivity of a network of ENZ nanostructures created in a random metallic structure. From the results of Figure 6, we observed that this approach allows for strong localization of optical radiation in nanoscale regions located well outside the metal, completely absorbing incoming optical photons in a specific bandwidth (Figure 6j and 6k). This approach can potentially enhance the absorption power of ultra-thin absorbers, which can take advantage of the formation of localized spots and harvest a significant portion of light energy in nm-thick film structures. The current photovoltaic technology employs Si absorbers of ~100 μm thickness, whereas other solution-processed materials with high manufacturability and low cost, such as quantum dots, require film thickness >1 μm to efficiently absorb all incoming photons. Our metastructures can considerably scale down these thicknesses, stimulating new research aimed at developing innovative materials for renewable energy harvesting.
