# Supplementary material for: Scalable, ultra-resistant structural colors based on network metamaterials
Source: Light Sci Appl. 2017 May 5;6(5):e16233–. doi: 10.1038/lsa.2016.233 (PMC6062193; doi:10.1038/lsa.2016.233)
Supplement: Supplementary Information [file lsa2016233x1.pdf]

# Supplementary Information for
# Scalable, ultra-resistant structural colors based on network metamaterials

Henning Galinski, Gael Favraud, Hao Dong, Juan S. Totero Gongora, Grégory Favaro, Max Döbeli, Ralph Spolenak, Andrea Fratalocchi, Federico Capasso

(Dated: March 15, 2017)

## Compositional and Structural Analysis

The growth of alumina (Al2O3) in/on the porous metal networks has been studied by focused ion beam (FIB) assisted scanning electron microscopy and Rutherford backscattering spectrometry (RBS). Ion beam polished cross-sections are shown in Supplementary Fig. S1. For an alumina thickness below 7 nm, the film grows conformally on the ligaments of nanoporous metal thin films. With increasing thickness the probability to close narrow regions in the open porous structure increases, resulting in a mixed growth regime within and on top of the porous film (Supplementary Fig. S1). Above an alumina film thickness of 18 nm, the porous network is sealed and the alumina grows only on top of the metal. This three-stage growth model has been verified using Rutherford backscattering spectrometry (RBS). The composition and thicknesses of samples with increasing $Al_2O_3$ layer are analyzed using a 2 MeV 4 He beam and a silicon PIN diode detector at 168 Celsius. The experimental data were fitted using the RUMP RBS simulation program [1]. Supplementary Fig. S2 shows a RBS spectrum for an 53 nm Al2O3 layer on top of a dealloyed Pt-YAl film. From the RUMP simulated spectra, we identify all critical parameters such as the thickness of the alumina layer (Supplementary Fig. S3) and the concentration of each material in the two layer system. The measured thickness and composition for all analyzed samples is given in Table I.
The choice of alumina is dictated by its mechanical robustness. Together a lightweight network architecture structure, this is the key for our metamaterial to achieve the impressive resistance observed in the scratch test of Fig. 3 of the main text. We have chosen the alloy presented here based on prior experiences and physical considerations. The atomic layer deposition (ALD) of $Al_2O_3$ takes place at 250 C, which demands for a temperature stable network. As low melting point materials such as Au, Ag already suffer from coarsening due to surface diffusion at these temperatures, we had to choose an alloy with a higher melting point and high thermal stability.

## Structural Coloration in Pt-Al networks

To demonstrate the effect of a different material on the network architecture and the structural coloration, we have chosen the binary alloy system Pt-Al. Pt-Al. Films have been deposited at room temperature by magnetron magnetron co-sputtering onto $SiO_2$/Si substrates that were pre-cleaned using isopropanol and acetone. The sputter power was set to 25 W for Pt and 250 W for Al, respectively. Subsequently, the films have been dealloyed in 2M NaOH at room temperature for 30 s and rinsed in deionised water afterwards. The morphological analysis of the samples was studied via scanning electron microscopy (SEM). An $Al_2O_3$ coating with a thickness gradient has been deposited via radio frequency (RF) sputtering at room temperature using an magnetron sputtering tool (PVD Products). The following deposition parameters have been used: base pressure 2:7 $10^{-7}$ Torr, Ar-pressure 4 mTorr, deposition power 70 W, deposition rate 0:5 nm $min^{-1}$, target $Al_2O_3$, no substrate rotation. The gradient in alumina thickness has been measured using ellipsometry on a $SiO_2$/Si wafer coated with $Al_2O_3$ gradient. Supplementary Fig. S9 reports the experimental findings for the Pt-Al system. The SEM micrographs in Supplementary Fig. S9a show a densification of the Pt-Al network compared to the Pt-Y-Al system. These network properties have been also reported, when using Pt networks as electrodes in fuel cells [2]. Similar to the Pt-Y-Al the deposition of an alumina subwavelength coating with gradual increasing thickness from 21 to 37 nm results in structural coloration (Supplementary Fig. S9b). The minimum observed in the reflectivity spectra shift as function of coating thickness d, as shown in Supplementary Fig. S9c. The wavelength shift of the reflectivity minimum as function of the alumina layer thickness d is lower than in the Pt-Y-Al case and here determined to be 6:6 nm $min^{-1}$. The colors achieved in the Pt-Al metallic network therefore do not extend the same range in the CIE chromaticity diagram (Supplementary Fig. S9d) as the Pt-Y-Al networks. This experiment shows that the network architecture has an important impact on the optical response of the system. A more detailed discussion on this topic goes outside the scope of this work and will be published in an upcoming paper.

## Scratch Resistance Tests

The adhesive failure of the network metamaterials has been analyzed by a series of scratch tests using a Nano Scratch Tester (Anton Paar TriSec). In these tests, we used a spherical diamond indenter with a radius of 2 $\mu$m is used (Supplementary Fig. S4a). For this analysis, all samples have been glued on a glass cover slip. The samples have been scratched with linearly increasing load (start load: 0:4 mN, end load: 15 mN) at a rate of

| ALD cycles | RBS thickness (nm) | Composition |
|---|---|---|
| 90 | $7 \pm 1$ | $Al_2O_3$ $Pt_{.18}$ $Y_{.54}$ |
| 135 | $12 \pm 1$ | $Al_2O_3$ $Pt_{.18}$ $Y_{.24}$ |
| 180 | $18 \pm 1$ | $Al_2O_3$ $Pt_{.18}$ $Y_{.24}$ |
| 225 | $24 \pm 1$ | $Al_2O_3$ - |
| 270 | $28 \pm 1$ | $Al_2O_3$ - |
| 360 | $33 \pm 1$ | $Al_2O_3$ - |
| 450 | $45 \pm 1$ | $Al_2O_3$ - |
| 540 | $53 \pm 1$ | $Al_2O_3$ - |

TABLE I. Measured $Al_2O_3$ coating thickness and composition as function of the number of growth cycles during deposition using atomic layer deposition.

29.2 mN min$^{-1}$. The scratch length was kept constant for all measurements at 300 nm, and the scratch speed was set to 10 $\mu$m s$^{-1}$. Supplementary Fig. S4b reports the mean residual depth measured as function of the stylus position and the applied load for a dense 300 nm thick Pt alloy thin film and 53 % porous Pt nanoscale networks coated with 28 and 53 nm alumina, respectively. The depicted curves are an average from 3 different scratch tests per sample. Although the density of the porous network metamaterials is significantly smaller than the dense Pt-alloy film, the critical load necessary to create an adhesion failure, i.e. delamination, is doubled from 6.2 mN to $\approx$ 13 mN. The network metamaterial with an 28 nm thick alumina coating (Supplementary Fig. S4b, orange curve) shows only a highly localised failure event and no total delamination of the coating.

## Trasformation optics of epsilon near zero (ENZ) regions

The calculation of the transformed refractive index in Fig. 1b can be accomplished through the form invariance of Maxwell equation under conformal mapping [3, 4]. This principle allows to transform spatial geometries into equivalent materials, and viceversa. Conformal mapping is developed by associating to each point of the original $(x, y)$ and transformed $(\psi, \phi)$ space a pair of complex numbers $z = x + iy$ and $Z = \psi + i\phi$, respectively, indicating with $z = \Omega(Z)$ the coordinate transformation relating the two spaces. This approach requires to express the coordinate transform by an analytic function $\Omega(Z)$. The transformation of coordinate in Fig. 5 of the main text is developed by generalizing the analysis of [5, 6] to arbitrary surface profiles. The mathematics of this analysis is quite involved and will be presented elsewhere. Once the transformation $\Omega(Z)$ is found, the transformed refractive $n(\psi, \phi)$ index is computed from the invariance of Maxwell equations [4] and reads as follows:

$$n(\psi, \phi) = \left| \frac{d\Omega(Z)}{dZ} \right|^2 . \quad (1)$$

## Light-matter interaction with alumina layers of different thicknesses

Supplementary Fig. S6 provides a pictorial view of the light matter interaction with our metamaterial structure, showing the trapping of backscattered waves that propagate at angles $\theta$ larger than the critical angle $\theta_c$ formed at the interface between alumina and air. These components generate a wave inside the alumina layer, which is sustained by Total Internal Reflection (TIR) at the air/alumina interface and by subsequent scattering at the alumina/metal layer side (Supplementary Fig. S6). The progressive scattering at the alumina/metal layer generates surface plasmon polariton waves, which are subsequently trapped in the equivalent epsilon-near-zero region of the metamaterial. The phase-matching between the TIR wave and the plasmon polariton is provided by the disordered metal, which act as a series of defects that furnish the additional momentum necessary for the interaction to take place (see [7], Chapter 3). This process of light matter interaction is quite complex and strongly wavelength selective, as both the additional momentum furnished by the metal and the spatial distribution of ENZ regions depend on the morphology of the disordered surface of the sample. The selectivity of this process is observed in Fig. 6a of the main text, which shows that among all backscattered waves, only a narrow band of wavelengths is able to strongly couple with surface plasmon polaritons and get trapped in the structure. Supplementary Fig. S8 presents FDTD computed electromagnetic energy distributions (Supplementary Fig. S8b-d) obtained by monochromatic excitations with wavelength corresponding to different reflectivity minima observed in the material response with increasing alumina thicknesses (Supplementary Fig. S8a). From panels b-d of Supplementary Fig. S8, we observe that different alumina thicknesses lead to the localisation of energy in the same set of epsilon near zero regions. The simplest condition for this outcome is that in all these cases the set of localised plasmon polaritons is formed by the TIR wave that corresponds to the same solution propagating in the rescaled structure, which leads to the same phase-matching condition. This is observed when:

$$\frac{\Delta d}{d_0} = \frac{\Delta \lambda}{\lambda_0}, \quad (2)$$

being $\lambda_0$, $d_0$ the wavelength and thickness related to one reflectivity minimum, respectively. This results from the scaling invariance of Maxwell equations: if we consider a slab waveguide and we change the thickness $d$, we obtain the same solution by shifting the wavelength in such a way that the ratio $\frac{d}{\lambda}$ is constant. Supplementary Eq. (2) is then obtained by setting the total differential of $\frac{d}{\lambda}$ to zero:

$$\Delta \left( \frac{d}{\lambda} \right) = \frac{\Delta d}{\lambda_0} - \frac{d_0}{\lambda_0^2} \Delta \lambda = 0. \quad (3)$$

This analysis provides an intuitive picture to establish a simple relationship between the wavelength shift $\Delta\lambda$ of the reflectivity minimum and the alumina layer thickness $d$: $\Delta\lambda = \frac{\lambda_0}{d_0}\Delta d$. For subwavelength coatings with $d \ll \lambda$, the wavelength shift $\Delta\lambda$ can be quite large ($\approx 10$ for coatings that are one order of magnitude smaller that visible wavelengths, in accordance with our measurements and FDTD), allowing the observation of a large variety of colours from extremely thin structures. The analysis of this section allows also to explain the absence of colours in the planar (i.e., non dealloyed) samples. When light is side coupled to these systems, in fact, it does not possess sufficient momentum to excite surface plasmon polariton waves [7], and the resulting reflectivity spectra do not show any special signature of light trapping or colour formation.

[1] Doolittle, L. & Thompson, M. Rump rbs simulation program. *(Materials Science and Engineering Department, Bard Hall, Cornell University)* (1985). URL `http://www.genplot.com/`.

[2] Ryll, T. *et al.* Dealloying of platinum-aluminum thin films: Electrode performance. *Phys. Rev. B* **84**, 184111 (2011).

[3] Pendry, J. B., Schurig, D. & Smith, D. R. Controlling electromagnetic fields. *Science* **312**, 1780–1782 (2006).

[4] Leonhardt, U. Optical conformal mapping. *Science* **312**, 1777–1780 (2006).

[5] Vandembroucq, D. & Roux, S. Conformal mapping on rough boundaries. ii. applications to biharmonic problems. *Phys. Rev. E* **55**, 6186–6196 (1997).

[6] Vandembroucq, D. & Roux, S. Conformal mapping on rough boundaries.mi. applications to harmonic problems. *Phys. Rev. E* **55**, 6171–6185 (1997).

[7] Maier, S. *Plasmonics: Fundamentals and Applications* (Springer, New York, 2010).

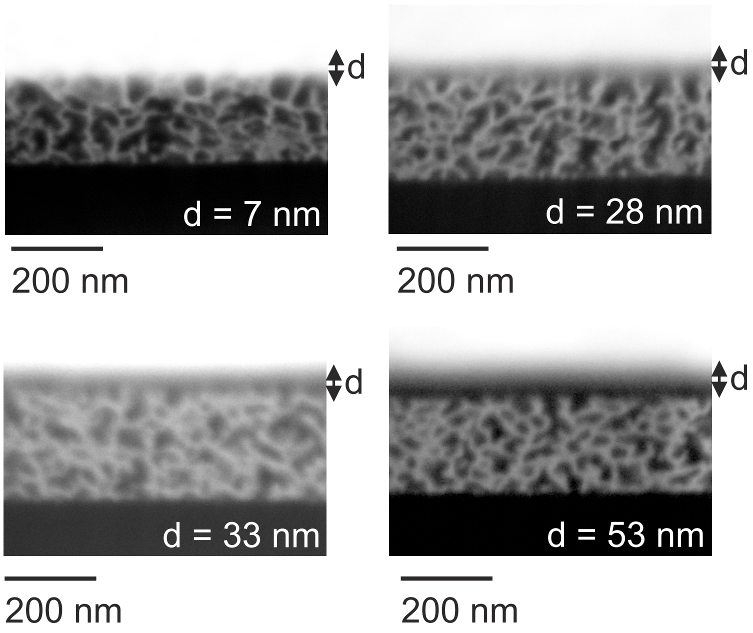


Supplementary Figure S1. Focused ion beam polished cross-section images of $Al_2O_3$ coated dealloyed $Pt_{.56}Y_{.26}Al_{.18}$ thin films depict the growth of the $Al_2O_3$ layer in and on top of the dealloyed thin film.

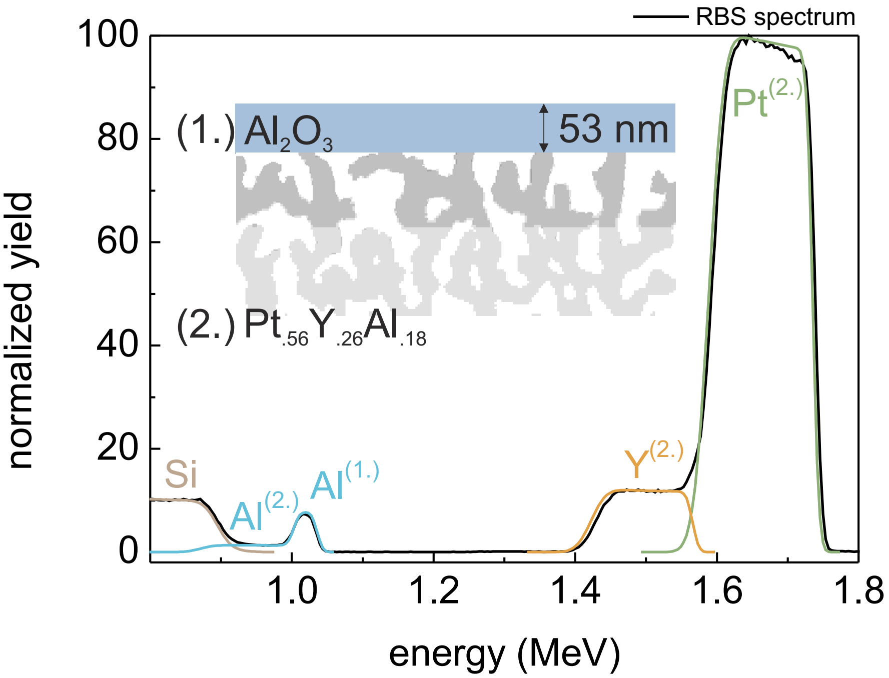


Supplementary Figure S2. Rutherford backscattering spectroscopy analysis of ALD grown of $Al_2O_3$ layer. The figure depicts a 2 MeV $4He^+$ RBS spectrum of dealloyed PtYAl thin films coated with 53 nm $Al_2O_3$ on a $Si_3N_4$/Si substrate. The RBS spectrum is compared to a RUMP simulation, depicting the components of the spectrum resulting from scattering by the single elements Pt,Y,Al and Si in the sample. The energy width of each element is proportional to the film thickness $h$, whereas the peak height, i.e. the yield $Q(E)$, is proportional to the concentration $X$ of the element at a given position in the sample.

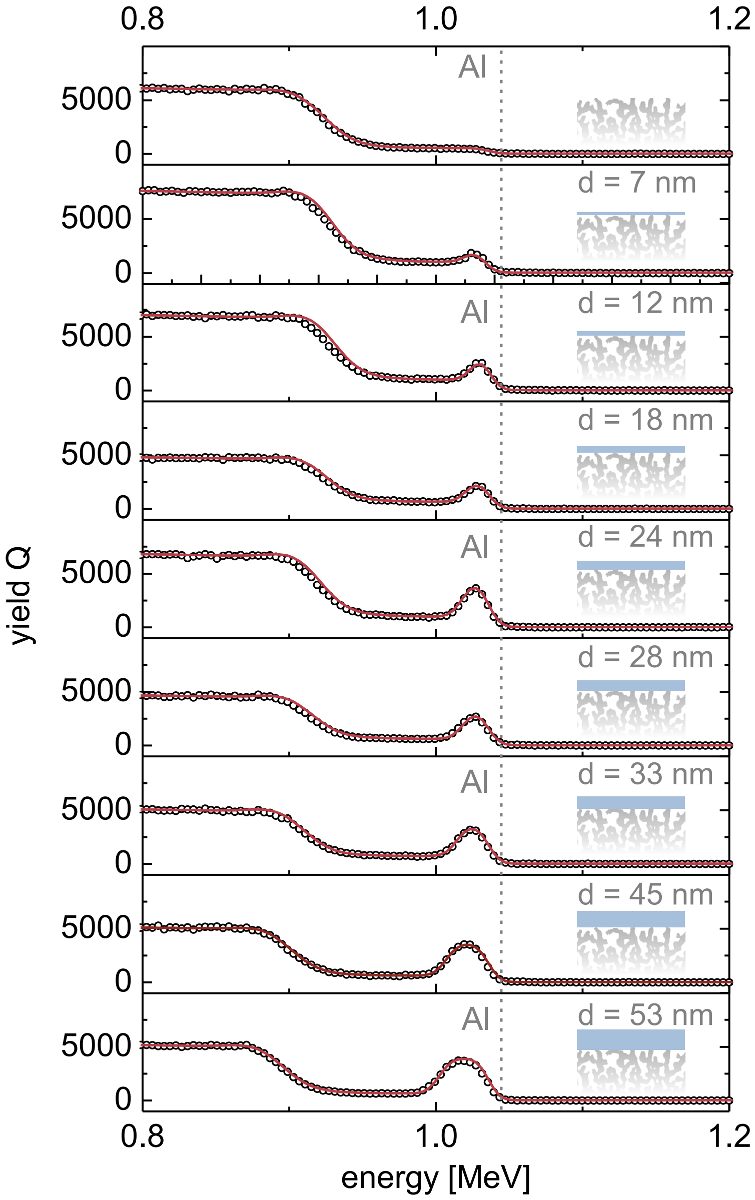


Supplementary Figure S3. $Al_2O_3$ layer growth captured by RBS. Rutherford backscattering spectroscopy (RBS) spectra of dealloyed Pt-Y-Al thin films coated with $Al_2O_3$ of different thickness are compared to simulated spectra (solid lines) using the RUMP RBS simulation program. The plot illustrates the growth of the $Al_2O_3$ layer on top of the dealloyed Pt-Y-Al thin films.

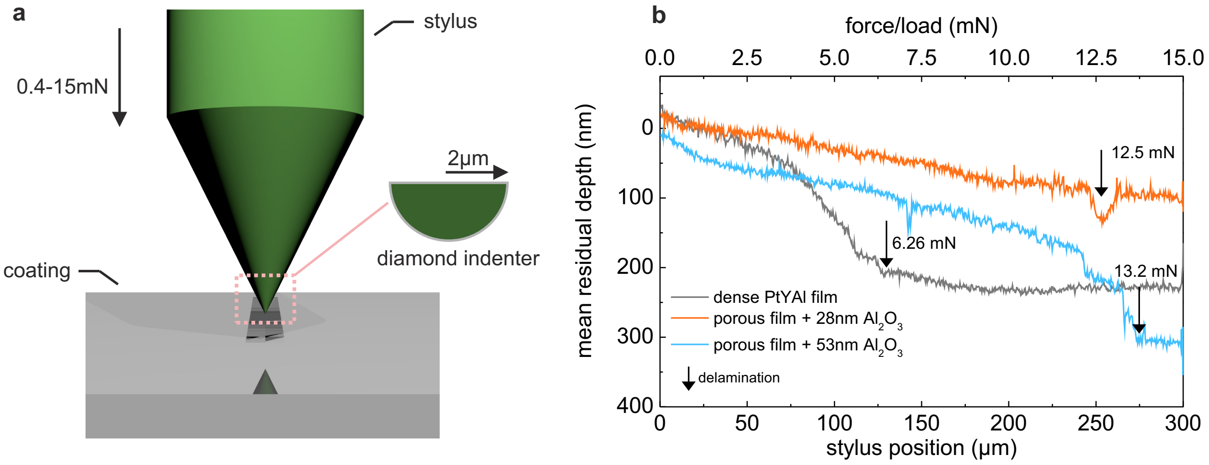


Supplementary Figure S4. Scratch resistance testing. (a) Schematic illustration of the stylus, equipped with a spherical diamond indenter, used for scratch testing. (b) Mean residual depth plotted as function of the stylus position and the applied load. The critical load causing the delamination of the coating is indicated by an black arrow.

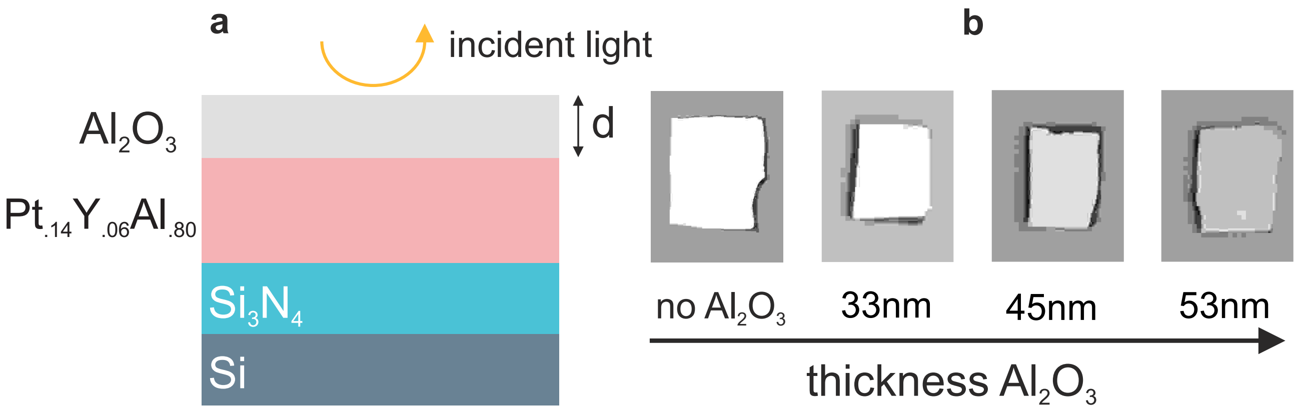


Supplementary Figure S5. ALD coating on a dense PtYAl film. (a) Schematic illustration of light interaction with an $Al_2O_3$ coated dense PtYAl thin film. (b) Photographs of PtYAl samples with no $Al_2O_3$ and different $Al_2O_3$ coatings deposited by atomic layer deposition on PtYAl thin films, illustrating that the present effect cannot be understood by interference or strong interference effects.

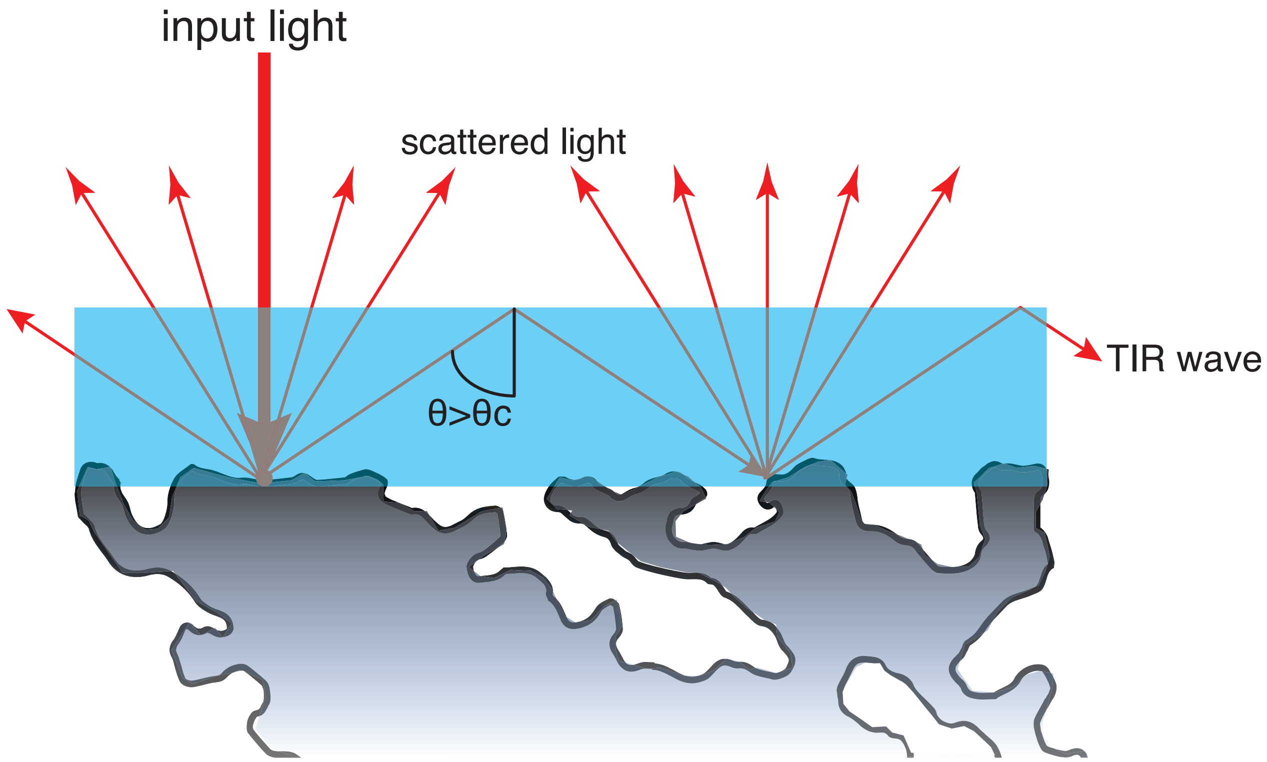


Supplementary Figure S6. Light matter interaction with the dealloyed metamaterial in the presence of alumina layer. The image shows a magnified picture of Fig. 6g of the main text, illustrating in more details the mechanism of formation of an energy flux inside the alumina layer sustained by Total Internal Reflection (TIR) of backscattered waves.

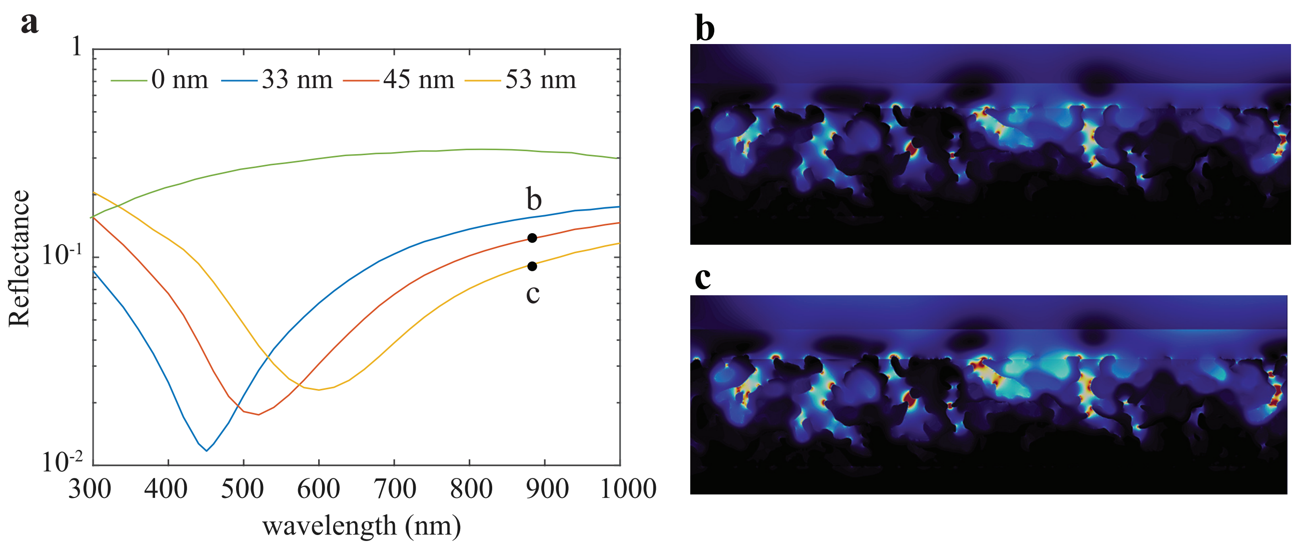


Supplementary Figure S7. Light-matter interaction with dealloyed metamaterial off resonance: FDTD results. (a) FDTD calculated reflectivity spectra for different $Al_2O_3$ thicknesses $d$. Panels (b-c) shows spatial distribution of averaged electromagnetic energy located away from the resonant point (i.e., reflectivity minimum) for different $Al_2O_3$ thicknesses (points b-c in panel a)

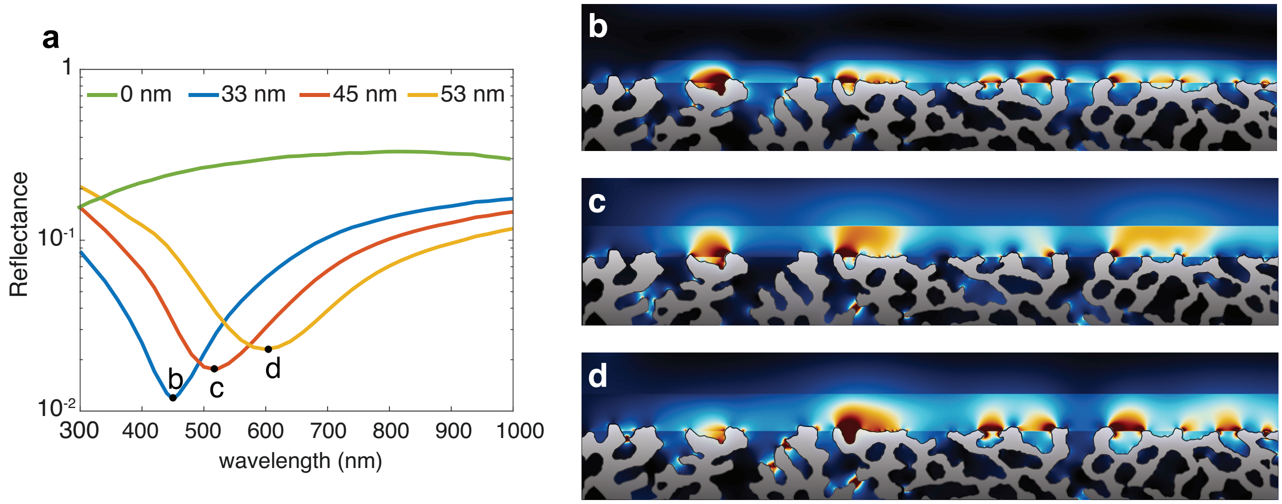


Supplementary Figure S8. Resonant light coupling with ENZ regions located in $Al_2O_3$: FDTD analysis. (a) FDTD calculated reflectivity spectra for different $Al_2O_3$ thicknesses $d$. Panels (b-d) shows spatial distribution of averaged electromagnetic energy at the resonant point (i.e., reflectivity minimum) for different $Al_2O_3$ thicknesses (points b-d in panel a)

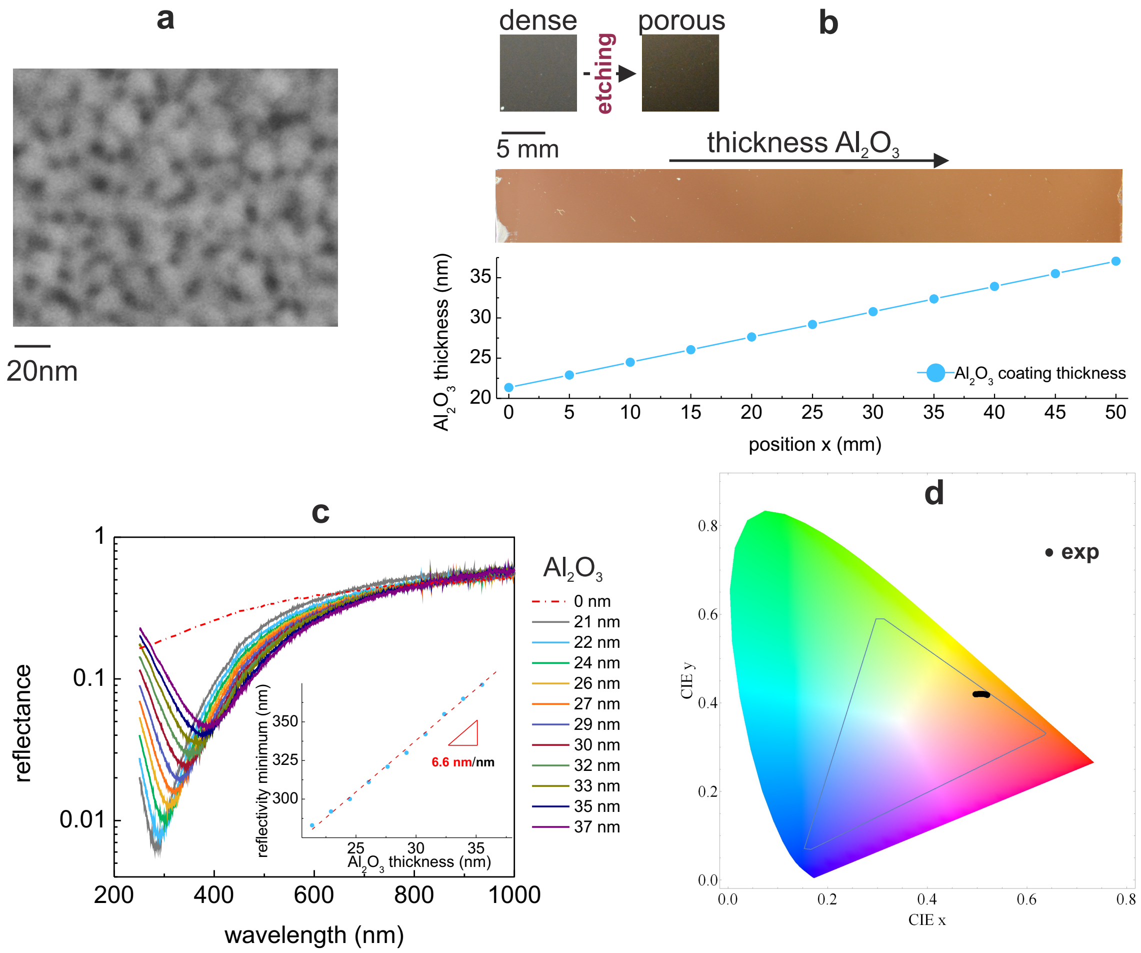


Supplementary Figure S9. Observation of structural coloration in Pt-Al metallic networks. (a) SEM top-view image of a dealloyed Pt-Al based network nanomaterial. (b) Photographs of as deposited, dealloyed and $Al_2O_3$ coated Pt-Al metamaterial-networks, illustrating the formation of color (yellow) with gradually increasing coating thickness. The photographs have beentaken under illumination from ceiling lights. The gradual increase of $Al_2O_3$ thickness has been determined by ellipsometry. (c) Experimental normal incidence reflectance spectra measured along the $Al_2O_3$ gradient. (d) Experimental structural color reported in a standard CIE 1931 (x,y) space, depicting all of the chromaticities visible to the average person. The RGB color space is marked by the triangle. The chromaticity is calculated directly from measured reflectance spectra (circles markers).
